# Distinct multitrophic biodiversity composition and community organization in a freshwater lake and a hypersaline lake on the Tibetan Plateau

**DOI:** 10.1016/j.isci.2024.110124

**Published:** 2024-05-27

**Authors:** Si-Yu Zhang, Qi Yan, Jindong Zhao, Yongqin Liu, Meng Yao

**Affiliations:** 1 School of Life Sciences, Peking University, Beijing 100871, China; 2 Institute of Ecology, College of Urban and Environmental Sciences, Peking University, Beijing 100871, China; 3 Center for Pan-Third Pole Environment, Lanzhou University, Lanzhou 730000, China; 4 State Key Laboratory of Tibetan Plateau Earth System, Resources and Environment (TPESRE), Institute of Tibetan Plateau Research, Chinese Academy of Sciences, Beijing 100101, China; 5 University of Chinese Academy of Sciences, Beijing 101408, China

**Keywords:** Ecology, Molecular biology, Aquatic biology

## Abstract

Alpine lakes play pivotal roles in plateau hydrological processes but are highly sensitive to climate change, yet we lack comprehensive knowledge of their multitrophic biodiversity patterns. Here, we compared the biodiversity characteristics of diverse taxonomic groups across water depths and in surface sediments from a freshwater lake and a hypersaline lake on the northwestern Tibetan Plateau. Using multi-marker environmental DNA metabarcoding, we detected 134 cyanobacteria, 443 diatom, 1,519 invertebrate, and 28 vertebrate taxa. Each group had a substantially different community composition in the two lakes, and differences were also found between water and sediments within each lake. Cooccurrence network analysis revealed higher network complexity, lower modularity, and fewer negative cohesions in the hypersaline lake, suggesting that high salinity may destabilize ecological networks. Our results provide the first holistic view of Tibetan lake biodiversity under contrasting salinity levels and reveal structural differences in the ecological networks that may impact ecosystem resilience.

## Highlights

• eDNA metabarcoding revealed the multigroup biodiversity of alpine lakes
• Freshwater and hypersaline lakes showed distinct community compositions
• Water and sediment biota differed significantly in each lake
• The hypersaline lake may have less stable ecological networks

## Graphical abstract

**Figure:**
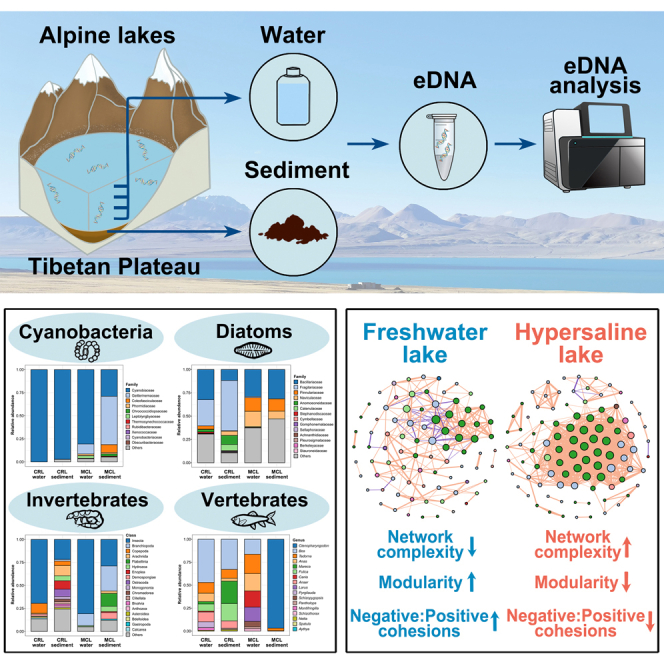

## Introduction

By integrating catchment-wide information and responding rapidly to environmental disturbances, lakes act as sensitive sentinel ecosystems for global climate change and regional ecosystem integrity.^1^^,^^2^ This sensitivity to change is even more pronounced in alpine lakes, because global warming has stronger impacts on high-altitude habitats and their associated biota.^3^^,^^4^ Changes in lake environments affect biodiversity, community composition, and species interaction patterns through direct and indirect mechanisms and may ultimately influence ecosystem function and essential services that lakes provide.^5^^,^^6^ Therefore, monitoring biodiversity patterns in alpine lakes is of paramount importance because such data reveal the mechanisms underlying ecological dynamics and provide a foundation for the design of informed conservation management strategies.^1^^,^^2^

Although the hydrological connectivity within lakes is generally good, lakes generally show significant environmental heterogeneity. Lakes encompass multiple spatial compartments with diverse pelagic and benthic habitats, and vertical zonation across the water column in deep lakes contributes additional heterogeneity.^7^^,^^8^ Adding to the spatial complexity of lakes, these compartments are linked by organism movement, biotic interactions, nutrient cycling, and energy flux, and hence a lake can be represented as a meta-ecosystem integrated through food web coupling and other ecological interactions.^9^^,^^10^ Previous research on lake biota often focused on a single habitat type,^11^^,^^12^ or in cases where multiple lake habitats were surveyed, only the microbial communities were investigated,^13^^,^^14^ which may have led to the biased understanding of lake biodiversity and community organization.

Because different organisms can show distinct habitat preference and environmental tolerance, a comprehensive analysis of biodiversity patterns calls for simultaneous examinations of multiple phylogenetic lineages and trophic groups to unravel the ecosystem-level factors contributing to community assembly and food web structure.^15^^,^^16^^,^^17^ However, simultaneous biodiversity surveillance across broad phylogenetic ranges and diverse lentic environments is rare, primarily due to the practical constraints associated with using traditional morphology-based survey methods. Assessments of multiple biological groups traditionally require a slew of sampling techniques, diverse taxonomy expertise, and intensive fieldwork and identification efforts. Rapid advances in molecular technology have given rise to highly efficient, cost-effective, and noninvasive biodiversity surveys based on environmental DNA (eDNA), i.e., organismal DNA present in environmental samples such as water, sediment, and soil.^18^^,^^19^ With the combined use of multiple primer sets and high-throughput sequencing, eDNA metabarcoding can be used to obtain data covering a wide taxonomic range or even complete “Tree of Life” biodiversity profiles, and hence enable the holistic reconstruction of the biological communities present in a given environment.^20^^,^^21^ More recently, eDNA has been integrated with ecological network analysis to reveal the organizational properties of ecological communities.^22^^,^^23^^,^^24^ Highly efficient eDNA bioassessments using multi-marker metabarcoding techniques facilitate the reconstruction of multitrophic ecological networks at the ecosystem level and substantially enhance the ability of researchers to unveil hidden biotic interactions and detect ecosystem changes.^25^^,^^26^^,^^27^

With over half of the total lake area in China and 1,424 lakes >1 km^2^, the Tibetan Plateau hosts the greatest density of alpine lakes in the world.^28^^,^^29^ Hailed as “the Water Tower of Asia”, this region provides essential water supplies and climate regulation to millions of people in South and Southeast Asia.^30^ However, the temperature of the Tibetan Plateau is rising at a rate 1.5 times the global average, which has led to considerable glacial recession, permafrost thawing, and changes in precipitation in recent decades.^28^^,^^31^ As a result, many lakes of the region are experiencing drastic alterations in water volume, thermal conditions, stratification regimes, hydrological dynamics, nutrient loading, and physiochemical properties.^32^^,^^33^^,^^34^ Moreover, most Tibetan Plateau lakes are endorheic lakes with moderate to high salinity.^35^ Detailed knowledge of biodiversity patterns in freshwater and saline lakes can therefore inform the future succession trends of aquatic communities and the consequent alterations to ecosystem processes. Yet biodiversity surveys of Tibetan lakes remain scarce, and little is known about their multitrophic community structure and ecological network properties.

**Figure 1:**
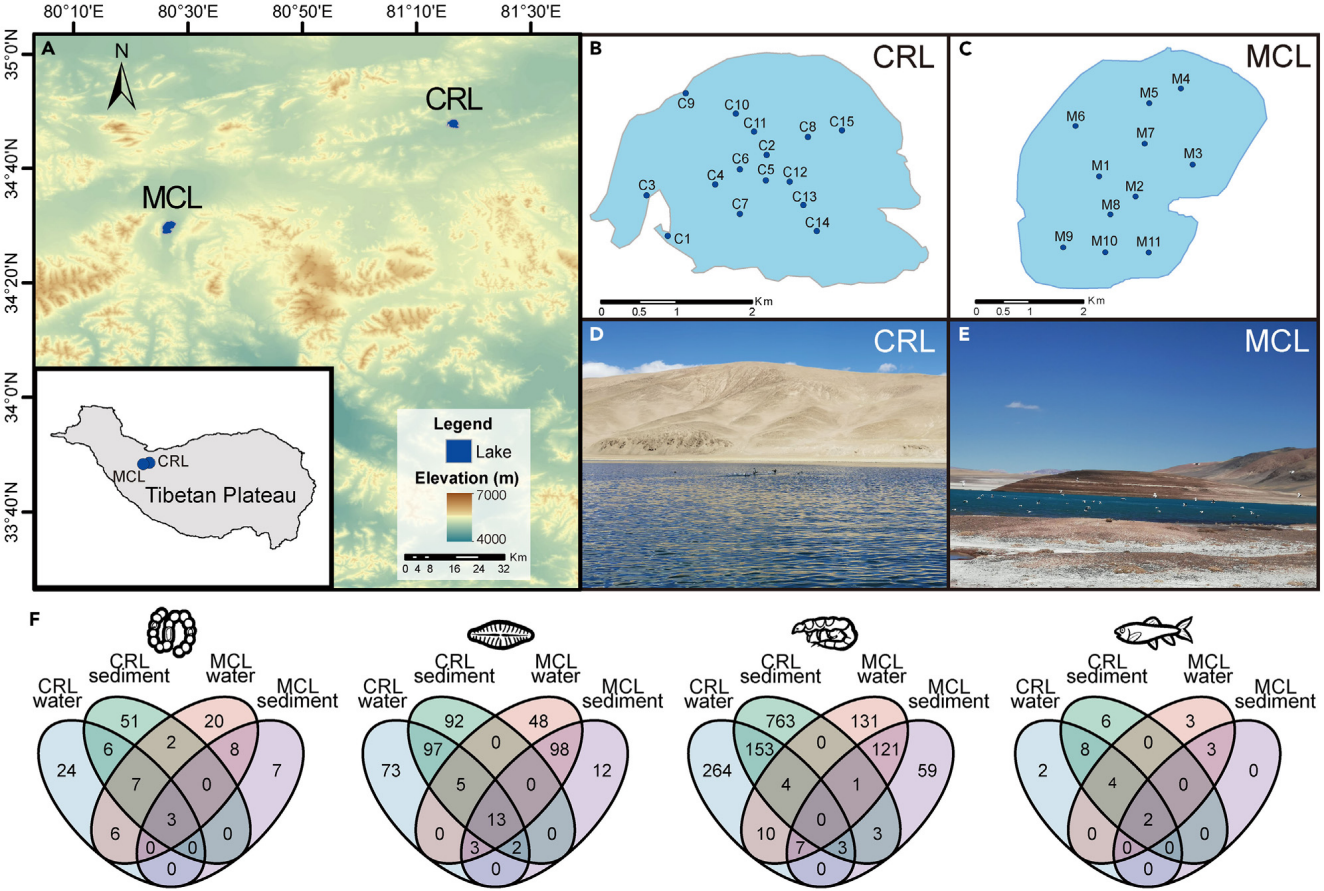
Study area, sampling design, and numbers of detected OTUs (A) Maps of the study lakes and sampling locations in (B) Chang-re Lake (CRL) and (C) Mang co Lake (MCL). Each location corresponds to a vertical series of water samples collected at various depths and a surface sediment sample. Photos of (D) CRL and (E) MCL lakeshore views are shown. (F) Venn diagrams showing the numbers of overlapping and distinct OTUs detected in the water and sediment samples from the two lakes. Panels (left to right) show the results for cyanobacteria, diatoms, invertebrates, and vertebrates.

In this study, we aimed to resolve the holistic biodiversity patterns and community network structure across the water column and in surface sediments of two high-altitude (approx. 5,000 m above sea level [a.s.l.]) lakes, one freshwater (Chang-re Lake; CRL) and one hypersaline (Mang co Lake; MCL), in Ngari Prefecture on the northwestern Tibetan Plateau (Figure 1). Ngari is one of the least explored regions of Tibet due to its extremely harsh natural environment. Using multi-marker metabarcoding of eDNA derived from water at multiple depths and from surface sediments, we analyzed the community composition of four taxonomic groups representative of different trophic levels, including cyanobacteria (prokaryotic primary producers), diatoms (eukaryotic primary producers), invertebrates (lower-level consumers), and vertebrates (top consumers). We then (i) investigated spatially explicit biodiversity and community compositions of different groups, (ii) assessed biodiversity patterns between the freshwater and hypersaline lakes, between water and sediment habitats, across the water depth gradient, and among water columns, and (iii) characterized and compared the structural properties of multitrophic cooccurrence networks both between lakes and between lake habitats.

## Results

### Multi-group biodiversity uncovered by eDNA

A total of 98.2 G sequencing data were generated from eight libraries (mean ± SD, 12.3 ± 2.8 G). After a series of quality filtering and bioinformatics processing steps (Table S2), an average of 10,000 to 40,000 reads were retained per PCR. The sequences altogether yielded 2,124 OTUs, including 134, 443, 1,519, and 28 OTUs for cyanobacteria, diatoms, invertebrates, and vertebrates, respectively (Table S3). The identified cyanobacteria OTUs belonged to 3 classes, 10 orders, and 18 families; diatoms to 3 classes, 9 orders, and 19 families; invertebrates to 17 phyla, 37 classes, and 33 orders; and vertebrates to 17 genera and 17 species (Data S2). Rarefaction curves of the recovered OTUs based on the number of reads per PCR indicated sufficient sequencing depth for the majority of samples (Figure S1).

Overall, greater taxa richness was detected from the freshwater (CRL) in comparison with the hypersaline lake (MCL) for each taxonomic group; 99, 285, 1,208, and 22 OTUs of cyanobacteria, diatoms, invertebrates, and vertebrates, respectively, were recovered from CRL samples, whereas 53, 181, 339, and 12 OTUs of the corresponding groups were detected in MCL. Only 13%, 5%, 2%, and 21% of the detected cyanobacteria, diatoms, invertebrates, and vertebrates, respectively, were shared between lakes, and a large fraction of each group was unique to one of the lakes (Figure 1F). For example, a number of known freshwater species, including several diatoms (e.g., *Cyclotella meneghiniana*, *Nitzschia palea*, *Nitzschia heufleriana*) (DiatomBase; https://www.diatombase.org) and invertebrates (e.g., oligochaete *Limnodrilus* sp.,^36^ ostracod *Ilyocypris* sp.;^37^ and fairy shrimp *Branchinecta orientalis*^38^), were only detected in CRL, whereas saline-adapted brine shrimp (*Artemia tibetiana* and *A*. *urmiana*) were only found in MCL. Among vertebrates, ruddy shelduck (*Tadorna ferruginea*), mallard (*Anas platyrhynchos*), Tibetan antelope (*Pantholops hodgsonii*), yak (*Bos grunniens*), and gray wolf (*Canis lupus*) were detected from both lakes. The Eurasian coot (*Fulica atra*), Northern pintail (*Anas acuta*), goose (*Anser* spp.), wigeon (*Mareca* spp.), and rufous-necked snowfinch (*Pyrgilauda ruficollis*) were unique to CRL, while two cyprinid fish endemic to the Tibetan Plateau, *Schizopygopsis stoliczkai* and *Schizothorax plagiostomus*, and the brown-headed gull (*Larus brunnicephalus*) were only detected in MCL.

### Multi-group biodiversity distribution within lakes

**Figure 2:**
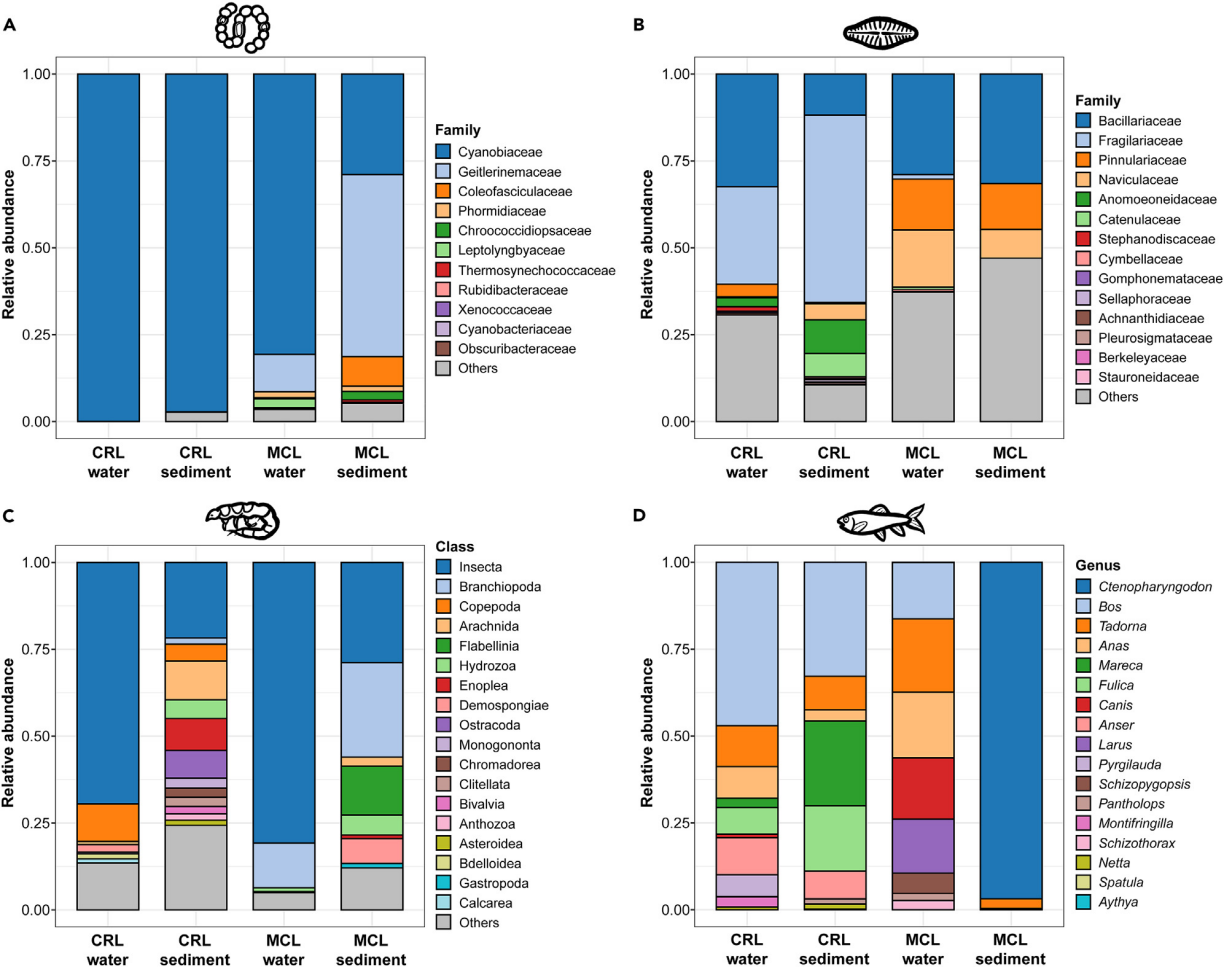
Community composition within each taxonomic group detected in water and surface sediments Relative read abundance (RRA) of OTUs is shown for (A) cyanobacteria, (B) diatoms, (C) invertebrates, and (D) vertebrates at the taxonomic level to which most OTUs of the group were assigned. Taxa are ordered from top to bottom by decreasing RRA across all samples. “Others” include OTUs of low relative abundance (RRA <0.01 for cyanobacteria and diatoms, and RRA <0.1 for invertebrates) and unassigned at the specified taxonomic level. CRL, Chang-re Lake; MCL, Mang co Lake.

When the results were analyzed by sample type within each lake, considerable differences were revealed between the biotic communities detected in water and sediment samples. For cyanobacteria, diatoms, invertebrates, and vertebrates, 16%, 41%, 13%, and 64% OTUs from CRL, and 21%, 63%, 38%, and 42% OTUs from MCL, respectively, were recovered in both water and sediments from the same lake, indicating considerable shared biodiversity between the two habitat types. Despite the possibility of eDNA dispersing away from where the originating organism resides, analysis of RRA-based data revealed clear compositional differences between water and sediment samples for each taxonomic group within each lake (Figure 2).

**Figure 3:**
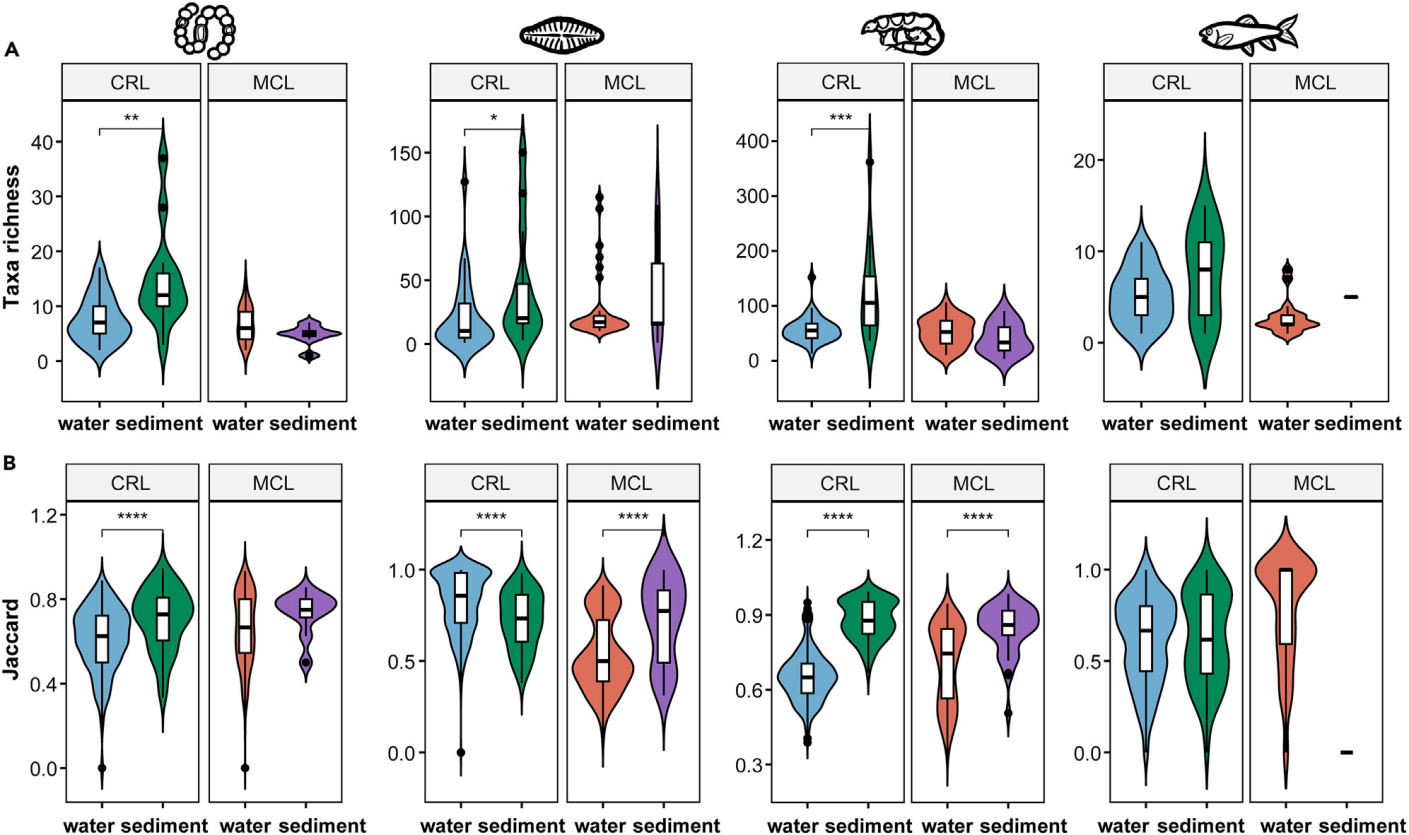
Comparisons of the α- and β-diversity of detected OTUs of four taxonomic groups between water and sediment samples Results are shown for (A) taxa richness; and (B) Jaccard dissimilarity. Panels (left to right) show the results for cyanobacteria, diatoms, invertebrates, and vertebrates. CRL, Chang-re Lake; MCL, Mang co Lake. Significant differences for pairwise comparisons determined by Wilcoxon rank-sum tests are shown: ∗, *p* < 0.05; ∗∗, *p* < 0.01; ∗∗∗, *p* < 0.001; ∗∗∗∗, *p* < 0.0001.

Comparison of the α-diversity of the recovered taxa per sample showed that a greater number of cyanobacteria, diatom, and invertebrate OTUs were detected in CRL sediment samples in comparison with CRL water samples, whereas no such difference was found for MCL samples (Figures 3A and S2). Comparison of the Shannon diversity between water and sediment samples showed largely comparable trends (Figure S3A). The between-sample difference (β-diversity; estimated by the incidence-based Jaccard dissimilarity) for cyanobacteria, diatoms, and invertebrates detected in sediments was generally greater than that of the same taxonomic group in water from both lakes, with the exception of diatoms in CRL, which showed the opposite pattern (Figure 3B). Analysis based on quantitative β-diversity (Bray-Curtis dissimilarity) produced results that were generally consistent with those based on the Jaccard dissimilarity (Figure S3B).

**Figure 4:**
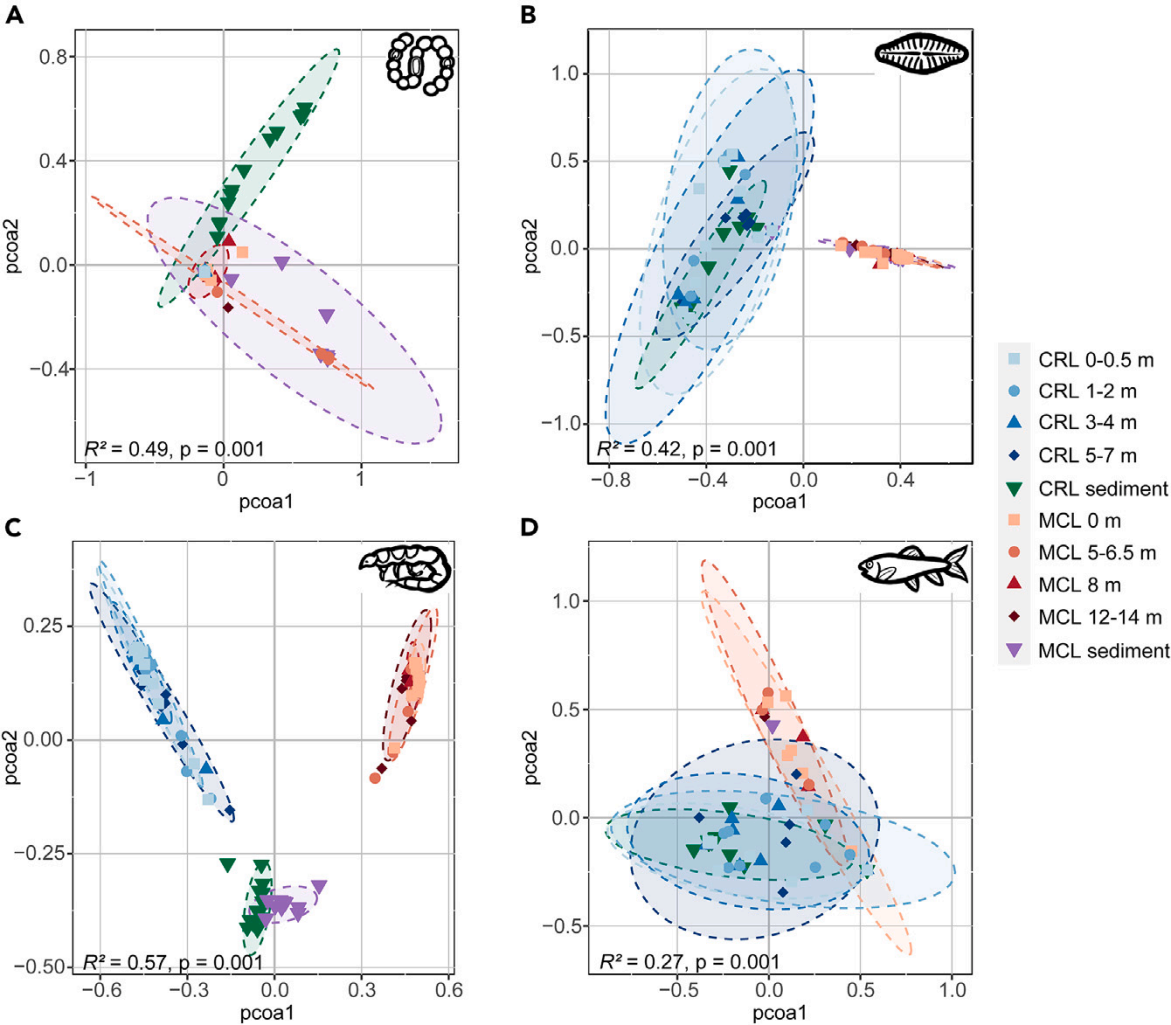
Principal coordinate analysis (PCoA) of the OTU compositions of the four taxonomic groups detected in water and sediment samples The PCoA was based on the quantitative Bray–Curtis dissimilarity index for (A) cyanobacteria, (B) diatoms, and (C) invertebrates, and on the qualitative Jaccard dissimilarity index for (D) vertebrates. CRL, Chang-re Lake; MCL, Mang co Lake. Results of PERMANOVA for the 10 sample groups are shown in each panel. See Table S4 for detailed results.

Physiochemical properties varied between the lakes, between water and sediment habitats, and vertically across the water column (Data S1); therefore, we assessed community compositional variations at these different scales. Compositional differences between communities were measured using quantitative Bray-Curtis dissimilarity for cyanobacteria, diatoms, and invertebrates, whereas qualitative Jaccard dissimilarity was used for vertebrates (see STAR Methods). PCoA of all samples demonstrated strong differentiation between the communities of cyanobacteria, diatoms, invertebrates, and vertebrates from both lakes, as well as the lesser disparity between the water and sediment communities within each lake (Figure 4). In contrast, communities of all groups detected in samples from different water depths largely overlapped (Figure S4). Statistical assessments using PERMANOVA were overall congruent with the PCoA results, with significant differences in the community composition of all taxonomic groups between lakes and between water and sediment samples within lakes (except for vertebrates) (all *p* < 0.01; Table S4). Further comparison between water depths revealed a significant community difference for diatoms in MCL, but not for any other group in either lake, which indicated limited stratification of biota in the water column. Additionally, PERMANOVA among different water columns showed significant horizontal community variations for cyanobacteria, diatoms, and invertebrates in CRL, but only for diatoms in MCL (Table S4).

### Characteristic taxa of different lakes and habitats

We first used SIMPER analysis to assess the contributions of individual OTUs to the RRA compositional differences between communities of each taxonomic group, both between the lakes and between habitats (water and sediments) (Table S5). We further determined the indicator OTUs characterizing different lakes and habitat types based on values (≥0.7) of the Occupancy and Specificity indexes (Table S6). The latter analysis generally identified more indicator OTUs than SIMPER for each group, (SIMPER results were subsets of OTUs identified by Specificity-Occupancy analysis), except for vertebrates. A number of OTUs were identified by both analyses. For cyanobacteria, various *Cyanobium* species accounted for large proportions of the community differences found both between and within lakes. In addition, a *Geitlerinema* OTU was characteristic of MCL (sediment in particular). For diatoms, community variations were mostly attributable to differences in the relative abundance of OTUs of the genera *Gedaniella*, *Nitzschia*, and *Pinnularia*. Notably, many indicator diatom OTUs found in MCL could not be identified at the genus or species level and thus lacked detailed taxonomic information (Table S6). The majority of the habitat-characteristic invertebrate OTUs were also only assigned at a high (Class) level, and the indicator OTUs were predominantly various insects. Notably, a brine shrimp species (*Artemia urmiana*) was characteristic of the hypersaline lake, MCL (Table S6). For vertebrates, differences in the relative eDNA abundance of yak, as well as that of several waterbirds (e.g., the mallard and ruddy shelduck), made large contributions to community variation between the lakes (i.e., more yak eDNA in CRL and more eDNA of the waterbirds in MCL; Table S5).

### Multitrophic cooccurrence networks

**Figure 5:**
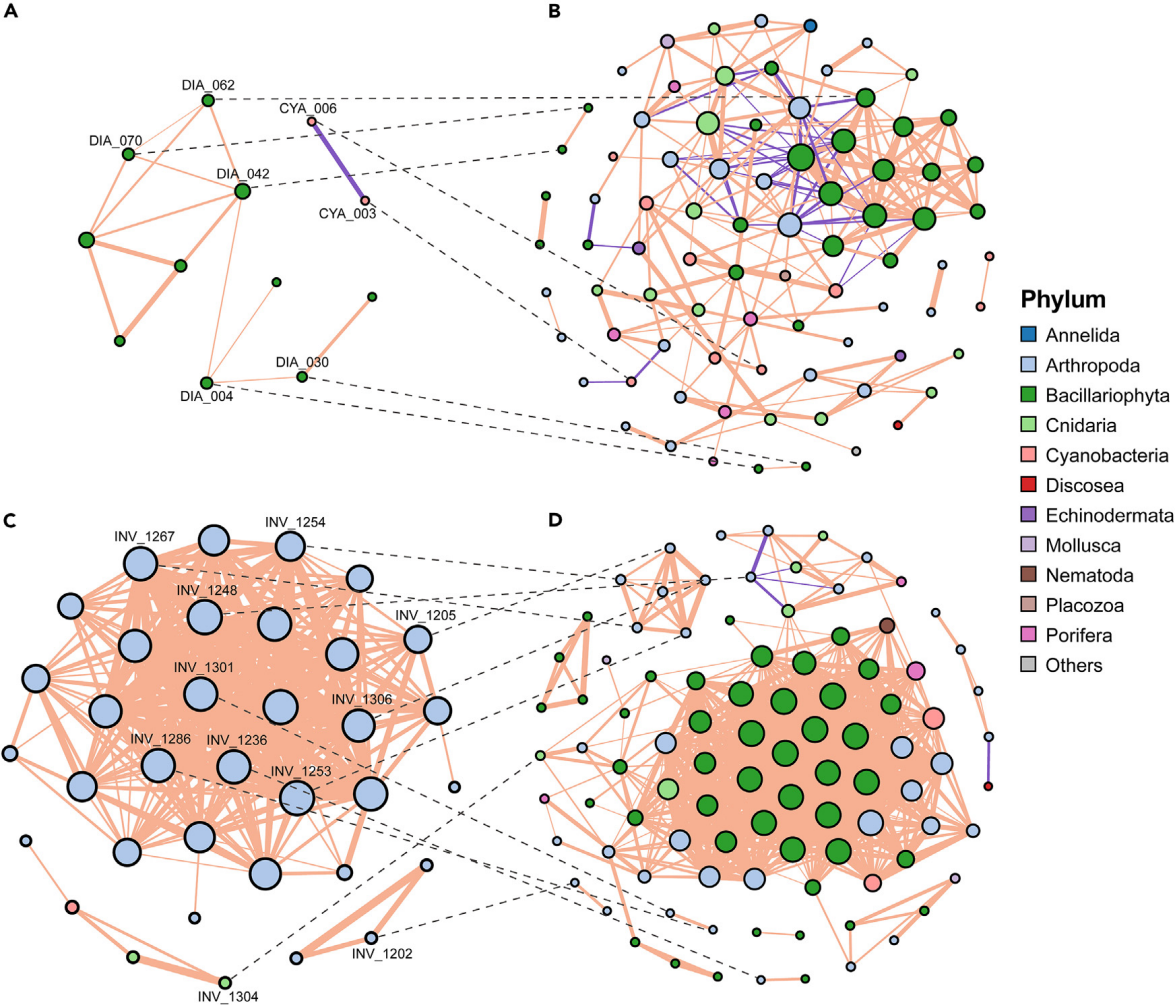
Cooccurrence networks of taxa detected in (A) water and (B) sediments of Chang-re Lake, and in (C) water and (D) sediments of Mang co Lake Nodes are OTUs showing significant correlations (Spearman’s |*r*| ≥0.75, *p* < 0.05) with other OTUs in each network. The color of each node indicates its phyla. Node size is proportional to the number of connections. Orange and purple connections depict positive and negative correlations, respectively, while the connection thickness represents correlation strength. Shared OTUs found in both the water and sediment networks of a particular lake are labeled and connected with dotted lines.

Based on Spearman correlations between the abundance (RRA) of OTUs across relevant samples, we constructed cooccurrence networks of multitrophic communities (including cyanobacteria, diatoms, and invertebrates) for water and sediment habitats of both lakes (Figure 5). For CRL, the water network was composed primarily of diatom OTUs and the sediment network contained diatoms and diverse invertebrate taxa. For MCL, the water network primarily consisted of artemia OTUs, while the sediment network contained many diatoms and diverse invertebrate taxa. The water and sediment networks shared 7 and 11 nodes (i.e., OTUs) in CRL and MCL, respectively. Although substantially more OTUs were detected in CRL in comparison with MCL across taxonomic groups, network properties showed a more complex and interconnected structure (greater numbers of nodes and correlations and higher connectedness and weighted degree) in MCL in comparison with that of CRL for the same habitat type, as well as for sediment in comparison with that of water within each lake (Figure 5; Table S7). In comparison with CRL, MCL had higher linkage density and clustering coefficient for each habitat (Table S7), which indicated greater network complexity and more closely associated network structure in MCL. However, the modularity (a measure of network compartmentalization) of CRL networks was greater than that of MCL networks for both water and sediment habitats, and the modularity of sediment networks was greater than that of water networks in each lake (Table S7).

**Figure 6:**
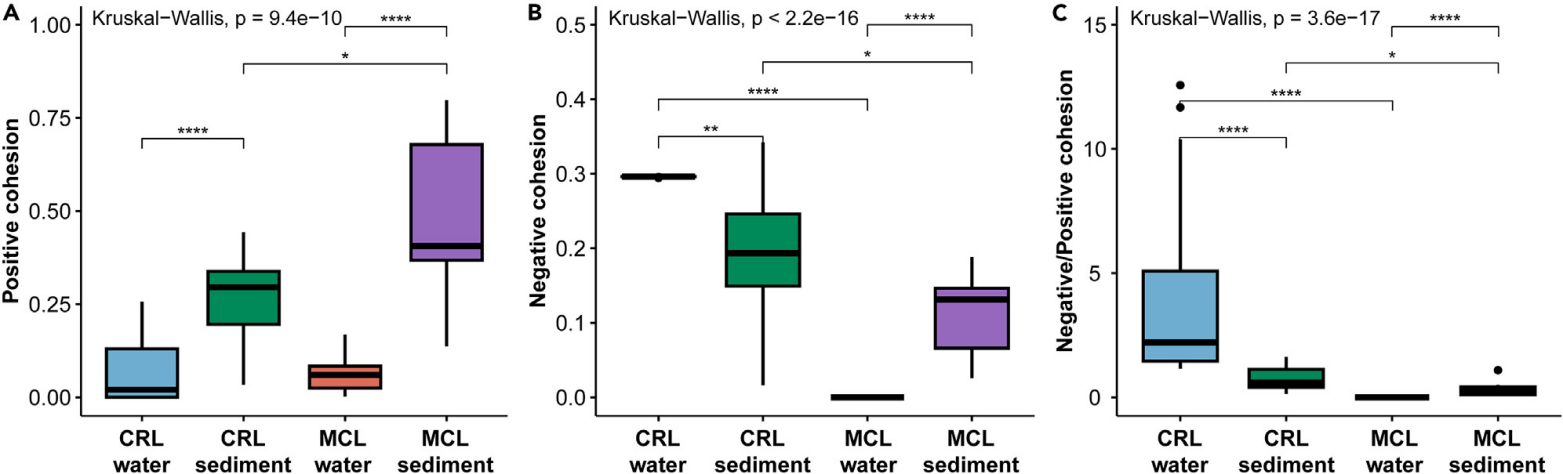
Comparisons of the structural characteristics of cooccurrence networks between water and sediment samples from the two lakes Results of the (A) positive, (B) negative, and (C) negative:positive cohesion values are shown. CRL, Chang-re Lake; MCL, Mang co Lake. Significant differences across the networks were assessed using the Kruskal-Wallis tests and indicated in each panel. Significant differences for pairwise comparisons determined by Wilcoxon rank-sum tests are shown: ∗, *p* < 0.05; ∗∗, *p* < 0.01; ∗∗∗, *p* < 0.001; ∗∗∗∗, *p* < 0.0001.

Comparison of the positive and negative correlations between nodes revealed that all four networks were dominated by positive correlations (Table S7). To assess the distribution of positive and negative correlations in the networks, we compared positive and negative cohesion values both between and within lakes. The number of positive cohesions detected in MCL was significantly greater than that of CRL for the sediment networks, whereas the number of negative cohesions was significantly lower in MCL in comparison with CRL for the networks of both habitat types, which led to a substantially greater negative:positive cohesion ratio in CRL in comparison with MCL (Figure 6; Table S7). Moreover, in both lakes, the number of positive cohesions in the sediment network was greater than that of the corresponding water network in the same lake, whereas a comparison of the number of negative cohesions between habitat types showed opposite patterns in the two lakes (Figure 6A). However, our analysis of negative cohesions and negative:positive cohesion ratio in different habitat types revealed divergent patterns in the two lakes. In CRL, both values were higher in the water network than in the sediment network, whereas in MCL, the values were higher in the sediment network compared to the water network (Figures 6B and 6C).

Potential keystone OTUs in each network were inferred by evaluating the topological parameters *Zi* and *Pi* for individual nodes (see STAR Methods). We identified six connectors (five diatoms and an OTU in the class Hydrozoa) in the CRL sediment network and one connector (diatom species *Nitzschia frustulum*) in the MCL sediment network (Table S8), hinting that these taxa may play organizing roles in their respective networks by connecting different modules. The water network was considerably less complex in comparison to the sediment network in each lake, and no connector or hub was identified in the water networks.

## Discussion

### Biodiversity in Tibetan freshwater and hypersaline lakes

The aquatic biota of the Tibetan Plateau is characterized by high endemism and carries unique biological and genetic signatures that reveal regional biogeographic history and demonstrate ecological adaption, thus providing an important source of data with significant value for research and conservation.^39^^,^^40^^,^^41^ However, the forbidding natural conditions of the Tibetan Plateau have proven to be a significant obstacle to systematic surveys of biodiversity in the region, especially for aquatic biota. Published surveys have covered only a small number of Tibetan lakes and have generally focused on a single taxon or organismal group in a particular habitat type (e.g., plankton, benthic macroinvertebrates, fish^42^^,^^43^^,^^44^^,^^45^), and thus have not fully captured the biotic communities present in lake ecosystems of the region. Our study provides the first holistic profiles of biodiversity in freshwater and hypersaline Tibetan lakes, which demonstrate that seemingly inanimate plateau lakes actually contain abundant life. Furthermore, water and surface sediment biota both made abundant and unique contributions to the total biodiversity of water ecosystems, demonstrating the importance of surveying both habitats to obtain a complete profile of contemporary aquatic biodiversity.^24^^,^^46^ Apart from aquatic species, eDNA of semi-aquatic and terrestrial animals, such as waterbirds, ungulates, and mammalian carnivores, was also recovered from water samples, demonstrating the capacity of the method to detect biota from both water and the surrounding land. Thus, eDNA-based analysis can serve as an efficient tool to document the landscape-level biodiversity of aquatic and terrestrial ecosystems.^47^^,^^48^

Freshwater and saltwater environments present distinct hydroosmotic and physiochemical conditions and harbor divergent species pools of various taxonomic groups. Indeed, salinity is a particularly strong driver of shifts in biotic community composition.^49^^,^^50^^,^^51^ We found significant community dissimilarity between the two lakes for all examined taxonomic groups, as well as a number of taxa indicative of each lake. For example, one of the characteristic taxa of MCL was brine shrimp, which are prevalent arthropods in Tibetan saline lakes.^2^^,^^52^ However, most of the identified indicator taxa either lacked fine taxonomic assignment (e.g., many Insecta OTUs) or had little ecological information available regarding their habitat preference (e.g., many cyanobacteria and diatom taxa). Interestingly, a cyanobacteria OTU indicator of MCL belonged to *Geitlerinema*, a genus that contains many freshwater species and was first identified in a freshwater sample from Shanghai, China in 2017.^53^ A recent study of *Geitlerinema* phylogeny revealed a subcluster containing free-living strains that occur in saline lakes across Russia.^54^ The *Geitlerinema* OTU identified in our study might be a member of that lineage, whose presence on the Tibetan Plateau was previously unknown. Additionally, *Nitzschia lembiformis*, a diatom species characteristic of MCL, was also found to be the most dominant diatom species in hypersaline ponds in Spain,^55^ suggest convergent filtering of microbial communities under similar environmental conditions.

The most pronounced difference in the community composition within each lake was found between water and sediment samples, as expected from the divergent life forms in the two distinct habitats. However, a lack of strong stratification across water depth for most groups (except for diatoms in MCL) suggests good mixing of lake water at the sampling time or frequent vertical movements of the biota. Unlike low-altitude lakes, the sustained low water temperature of Tibetan lakes may inhibit sufficient stratification even in deep lakes. In the horizontal direction, communities generally showed significant variations among water columns in CRL, while they did not show this variation in MCL (except for diatoms), which indicates that there may be stronger spatial environmental heterogeneity in CRL.^56^^,^^57^ A future study to assess various environmental parameters could further resolve how abiotic and biotic factors affect community structure and assembly within lakes.

### Community organization and water–sediment linkages

The structural properties (e.g., complexity, modularity, and nestedness) of ecological networks convey information beyond simple metrics of taxonomic diversity and have critical implications for species interactions, community stability, and ecosystem functioning.^26^^,^^58^^,^^59^^,^^60^^,^^61^ Thus, the characterization of ecological network organization is increasingly valued in community ecology and ecosystem assessments.^62^^,^^63^ Although cooccurrences between taxa do not necessarily imply functional relationships (e.g., predation, mutualism), strong correlations and network organization patterns revealed by ecosystem-scale analysis across multiple localities may offer valuable insight into ecological interactions and community structural characteristics. Several of our findings are worth noting. First, while the taxonomic diversity of all groups was higher in the freshwater lake, the hypersaline lake networks showed greater structural complexity and connectedness, which may be explained by strong environmental stressors (e.g., high salt concentrations and osmotic pressure) forcing tight associations among organisms. Furthermore, network modularity and negative:positive cohesion were both lower in the hypersaline lake networks. These network traits have been positively associated with ecological community stability in plant–pollinator and food web networks,^64^^,^^65^ as well as in microbial networks.^66^^,^^67^ Interestingly, a recent study of river food-web structure also showed decreased network modularity and negative:positive cohesion under persistent anthropogenic stressors.^68^ Thus, our results suggest that the hypersaline lake ecosystem may be more vulnerable to environmental disturbances and may show stronger responses to climate changes in the coming decades.

An additional notable finding that emerged from our analyses is the presence of distinct, but closely coupled, communities across water and surface sediment compartments within each lake. Pelagic and benthic biota in lakes are often regarded as distinct communities and studied in isolation. It is apparent that these habitats each represent disparate environmental conditions and host different organisms, as shown by the significantly different community compositions of water and sediment samples from the same lake for all examined taxonomic groups (Figure 4). However, many organisms occupy different habitats within lakes at different life cycle stages, during different seasons of the year, or even at different times during the day.^69^ For instance, it is common for phyto- and zooplankton species to have benthic life stages (e.g., as eggs, cysts), and for bottom-dwelling invertebrates to have free-swimming larva. Also, aquatic species can prey on organisms in the surface sediment, and vice versa.^9^ Therefore, ecological communities in the water and surface sediment habitats of a lake are bound by organism movements, biotic interactions, and nutrient cycling, and their networks are necessarily intertwined to constitute a within-lake meta-ecosystem.^8^^,^^9^ Further simultaneous assessments of various lake compartments and their ecological organization will shed light on how biotic community composition and interactions between communities modulate energy flux and biogeochemical cycling in lake ecosystems.

### Implications for future research

Rapid worldwide glacial melting causes the expansion of alpine lakes, which drives alterations in lake salinity and other physiochemical properties that affect species distribution pattern.^70^^,^^71^ Species composition variations in aquatic ecosystems driven by salinity and other environmental alterations may reshape trophic interactions and food-web structure, leading to changes in biogeochemical cycling and ecosystem functioning.^72^^,^^73^^,^^74^ Moreover, the rising temperature can alter lake thermal conditions, mixing patterns, and growing season duration. All of these alterations can further affect the abundance, composition, and dynamics of lake-dwelling communities.^34^^,^^75^ However, our understanding of biotic community organization and ecosystem processes on the Tibetan Plateau remains rudimentary and insufficient for reliable trend-prediction and informed management. eDNA-based biomonitoring can circumvent many of the obstacles associated with field sampling and specimen identification, allowing studies aimed at comprehensively understanding biodiversity and ecosystem organization in challenging environments such as the Tibetan Plateau. The multitrophic biodiversity pattern and ecological network architecture in Tibetan lakes revealed in this study lay the foundation for future investigations across spatiotemporal scales and environmental gradients to extend our knowledge of these vital systems and to guide conservation management in the face of global climate change.

### Limitations of the study

Despite the many advantages of eDNA biomonitoring, the effectiveness, and accuracy of DNA-based species identification are highly dependent on the taxonomic coverage and data quality of reference sequence databases.^76^ Since limited genetic characterization of biota has been carried out in Tibetan Plateau ecosystems, regional endemic species are generally underrepresented in publicly available sequence reference databases.^77^^,^^78^ Therefore, the biodiversity identified via eDNA analysis of samples collected from Tibetan Plateau ecosystems likely underestimates the actual species diversity. Continued effort to expand genetic database coverage for Tibetan biota is of paramount importance for improving understanding of the region’s biodiversity and for implementing routine eDNA-based biomonitoring to trace the range shifts and community dynamics of endemic biota.

Alpine lakes are under strong influences of plateau environments and exhibit drastic spatial and temporal dynamics in their thermal condition, hydrological regime, circulation pattern, and nutrient availability.^32^^,^^79^^,^^80^ Accordingly, various lake biota may respond to these influences in terms of species composition, community structure, and biotic interactions. This study captured a snapshot of biodiversity patterns in two lakes on the Tibetan Plateau. Extensive research that encompasses broad geological ranges and temporal scales, as well as various physiochemical and hydrological properties, is crucial for understanding the spatial and seasonal variations of Tibetan Plateau lake biodiversity and making informed predictions about future ecosystem trends.^2^^,^^81^

## STAR★Methods

### Key resources table

**Table:** 

REAGENT or RESOURCE	SOURCE	IDENTIFIER
**Biological samples**		

40 water samples	Chang-re Lake, Ngari Prefecture, Northwestern Tibetan Plateau	N/A
14 sediment samples	Chang-re Lake, Ngari Prefecture, Northwestern Tibetan Plateau	N/A
36 water samples	Mang co Lake, Ngari Prefecture, Northwestern Tibetan Plateau	N/A
11 sediment samples	Mang co Lake, Ngari Prefecture, Northwestern Tibetan Plateau	N/A

**Chemicals, peptides, and recombinant proteins**		

Bovine serum albumin (BSA)	AMRESCO	Cat#0903
Premix Ex Taq	Takara Bio	Cat#RR902A
RNase-free water	Takara Bio	Cat#9012
Agarose	Biosharp	Cat#BS081
GelStain	TransGen Biotech	Cat#GS101
BM2000 DNA Marker	Biomed	Cat#MD101

**Critical commercial assays**		

DNeasy PowerSoil Pro Kit	QIAGEN	Cat#47016
MiniBEST DNA Fragment Purification Kit	Takara Bio	Cat#9761
47mm 0.2-μm polycarbonate membranes	Millipore	Cat#GTTP04700
Next-generation sequencing	Novogene	https://www.novogene.com/us-en/technology/platforms/#sequencing

**Deposited data**		

Raw sequencing data	This paper	BioProject: PRJNA1062616; Accessions: SRR28468975 – SRR28468982

**Oligonucleotides**		

CYA359F/CYA784R primers – Forward: 5′-GGGGAATYTTCCGCAATGGG-3′; Reverse: 5′-ACTACWGGGGTATCTAATCCC-3′	Monchamp et al.^82^	N/A
708F_2/R3_1 primers – Forward: 5′-AGGTGA AGTTAAAGGTTCATACTTDAA-3′; Reverse: 5′-CCTTCTAATTTACCAACAACTG-3′	Vasselon et al.^83^	N/A
BF1/BR2 primers – Forward: 5′-ACWGG WTGRACWGTNTAYCC-3′; Reverse: 5′-TCDGGRTGNCCRAARAAYCA-3′	Elbrecht and Leese^84^	N/A
Tele02-F/R primers – Forward: 5′-AAAC TCGTGCCAGCCACC-3′; Reverse: 5′-GGGTATCTAATCCCAGTTTG-3′	Taberlet et al.^85^	N/A

**Software and algorithms**		

OBITools3	Boyer et al.^86^	https://git.metabarcoding.org/obitools/obitools3/
SUMACLUST	Mercier et al.^87^	http://metabarcoding.org/sumaclust
DADA2	Callahan et al.^88^	https://benjjneb.github.io/dada2/
RDP Classifier v.2.14	Wang et al.^89^	https://sourceforge.net/projects/rdp-classifier/
BOLDigger	Buchner and Leese^90^	https://github.com/DominikBuchner/BOLDigger
BLAST	Altschul et al.^91^	https://blast.ncbi.nlm.nih.gov/Blast.cgi
R v.4.2.1	R Core Team^92^	https://cran.r-project.org/bin/windows/base/
Gephi v.0.9.7	Bastian et al.^93^	https://gephi.org/

### Resource availability

#### Lead contact

Further information and requests for reagents should be directed to and will be fulfilled by the lead contact, Meng Yao (yaom@pku.edu.cn).

#### Materials availability

This study did not generate new unique reagents.

#### Data and code availability

• The sequencing data are available in the National Center for Biotechnology Information (NCBI) Sequence Read Archive (SRA) database, with the accession numbers listed in the key resources table.
• This paper does not report original code.
• Any additional information required to reanalyze the data reported in this paper is available from the lead contact upon request.

### Method details

#### Study sites and sampling

The Tibetan Plateau is the largest (2 million km^2^) and highest (average altitude above 4,500 m a.s.l.) plateau on Earth. Chang-re Lake (CRL; 34°47′ N, 81°16′ E) and Mang co Lake (MCL; 34°29′ N, 80°26′ E) are located in Rutok County of Ngari Prefecture, with a water level of about 5,000 m a.s.l. The area has a typical subfrigid semi-arid climate, with accumulation of white solid calcium carbonate in soil. The area has a very low human population density (average 0.14/km^2^; 2021 census), and the local economy mainly relies on livestock (yaks, horses, sheep, and goats) farming. CRL is a freshwater lake with a surface area of 7 km^2^ and a maximum depth of 7 m, and MCL is a hypersaline (average 83 practical salinity units [psu]; Data S1) lake with a surface area of 12.4 km^2^ and a maximum depth of 14 m. Both lakes are perennially characterized by low temperature, low productivity and limited nutrient availability, and each lake is fed by frozen soil melt and groundwater inflow. According to calculations based on the ERA5 database,^94^ the annual average temperature of CRL is approximately −6.8°C, whereas that of MCL is −8.9°C. The lakes both receive relatively little precipitation; the annual rainfall at CRL is 7.67 mm, whereas that of MCL is 8.68 mm. Little vegetation was found on the shore of either lake, and neither lake showed detectable macrophyte growth at the time of sampling.

In September 2020, water at four depth ranges and surface sediment samples were collected along 12 and 11 vertical columns from CRL and MCL, respectively. Additional sediment samples were at three locations from CRL (Figure 1; Data S1). One 1-L water sample was taken at 0–0.5, 1–2, 3–4, and 5–7 m of each column in CRL, and 500-mL water samples were collected at 0, 5–6.5, 8, and 12–14 m of each column in MCL (Data S1), using a Schindler hydrophore. Water samples were individually prefiltered through 20-μm bolting silks to remove large plankton and particulate matter, then filtered through 0.2-μm polycarbonate membranes (47 mm, Millipore, Burlington, Massachusetts, USA). One surface sediment sample (about 0.5 g) was collected from each sampling column in each lake. A total of 40 water and 14 sediment samples were collected from CRL, while 36 water and 11 sediment samples were obtained from MCL. All filter membranes and sediment samples were transported frozen to the laboratory and stored at −20°C for further DNA extraction. Physiochemical parameters, including water temperature, salinity, pH, conductivity, and total dissolved solids, were measured on site using a handheld YSI EXO2 Multiparameter Sonde (YSI, Yellow Springs, Ohio, USA).

#### Laboratory procedures

The DNA extraction and PCR preparation steps were performed in a dedicated eDNA processing room that was separated from the laboratories used for PCR and post-PCR procedures. The experimental environment was kept clean with bleach and ultraviolet light. Total DNA was extracted from each water and sediment sample using the DNeasy PowerSoil Pro Kit (Qiagen, Hilden, Germany) following the standard manufacturer’s protocols. We conducted separate PCR assays to amplify the DNA of each of the four target groups. Specifically, the barcodes residing in the 16S rRNA gene for cyanobacteria (CYA), the rbcL gene for diatoms (DIA), the COI gene for invertebrates (INV), and the 12S rRNA gene for vertebrates (VER) were amplified using primer sets CYA359F/CYA784R, 708F_2/R3_1, BF1/BR2, and Tele02-F/R, respectively (Table S1). These primer sets have been verified for their capacities to resolve biodiversity within the designated taxonomic groups.^16^ Each PCR consisted of 2.5 μL 5-fold diluted eDNA template (to reduce PCR inhibitors), 0.2 μM each of forward and reverse primers, 0.4 mg/mL bovine serum albumin, 12.5 μL 2 × Premix Ex Taq (Takara Bio Inc., Kusatsu, Japan), and 8.5 μL RNase-free water (to a total volume of 25 μL). Two cytosines and a unique 8-bp sequence tag were added at the 5**′** end of both the forward and reverse primers to allow sample identification following sequencing.^95^ Additional information regarding the primers and PCR programs is included in Table S1. Triplicate PCRs were conducted for each DNA extract, and three PCR negative controls (containing all other reagents except the DNA template) were also included in each PCR run. PCR products were checked by 1.5% agarose gel electrophoresis and purified using the MiniBEST DNA Fragment Purification Kit (Takara Bio, Shiga, Japan). Separate libraries (8 total) for each lake and each primer set were constructed using PCR products pooled in equal volumes. DNA concentrations were estimated on a NanoDrop 2000 spectrophotometer (Thermo Scientific, Waltham, Massachusetts, USA). Paired-end sequencing (2 × 250 bp for CYA, DIA, and INV libraries; 2 × 150 bp for VER libraries) was performed on a NovaSeq 6000 platform (Illumina Inc., San Diego, California, USA) at Novogene Sequencing Services (Beijing, China).

#### Sequence processing

OBITools3 package was used to filter and process raw Illumina-sequencing data.^86^ Briefly, full sequences were recovered from forward and reverse reads and assigned to corresponding samples by tags. Sequences with a low alignment score were removed. Reads were dereplicated into unique sequences. Sequences with fewer than 10 reads per library and a length less than the minimum length for each barcode were removed. Putative PCR and sequencing errors were removed using *obi_clean* (r = 0.1). The sequences of both lakes were combined for each primer set and clustered into preliminary operational taxonomic units (OTUs) at a 97% (for cyanobacteria, diatom, and invertebrate sequences; following previous studies^16^^,^^96^) or 98% (for vertebrate sequences) similarity threshold using the SUMACLUST package.^87^ A higher clustering threshold was used for vertebrate sequences to retain more sequence variants,^97^^,^^98^ which were then individually matched to the GenBank database and manually examined to determine their taxonomic identity (see below). The step-by-step process and results are included in Table S2.

For cyanobacteria and diatoms, taxonomic annotation was performed using the RDP naïve Bayesian classifier implemented in the *assignTaxonomy* function in the DADA2 package^88^ against the SILVA v.138^99^ or Diat.barcode v.10^100^ database, respectively. The minimum bootstrap value was set to 75, and species-level assignment was further refined using the *addSpecies* function. Taxonomic assignment for invertebrates was performed by using both the *classifier.jar* function in the RDP Classifier v.2.14 program^89^ and the JAMP pipeline in the BOLDigger package,^90^ against the MIDORI2 unique COI v.GB256 database^101^ and Barcode of Life Data System (BOLD; https://boldsystems.org/), respectively. The bootstrap threshold was set to 100 for the species level and set to 75 for other taxonomic levels. Two database annotation results were integrated, and the highest resolution for each OTU was chosen as the best hit. Results of invertebrates at the species level were additionally checked by the Basic Local Alignment Search Tool (BLAST),^91^ against the GenBank nucleotide database. Taxonomy was assigned to vertebrate OTUs using BLAST and the nucleotide database in GenBank. The matched results with the highest Max scores and >95% similarity at 100% coverage were retained. Vertebrate OTUs were further merged and refined based on local species records^102^ following criteria described previously.^16^^,^^103^ Briefly, sequences with similarity between 95% and 98% were assigned to the corresponding genus level. If the query sequence matched a single local species with ≥98% similarity, the species was assigned. If the query sequence matched more than one local species with ≥98% similarity, the lowest taxonomic level that included all local species was assigned.

Only sequences assigned to the corresponding taxonomic groups were retained for further analysis. Negative control PCRs generally produced low read counts (mean = 0.22–24 in each library). To reduce potential cross-sample contamination, the maximum count of each OTU in the negative control PCRs was subtracted from the read count in each sample PCR. OTUs with <5 reads per PCR were removed to reduce cross-contamination and tag-jumps.^104^ Rarefaction curves were constructed by the *rarecurve* function of the R package vegan^105^ to evaluate the effect of sequencing depth on OTU detection. Based on the rarefaction results, PCRs with <100 reads were discarded for cyanobacteria, diatom, and vertebrate OTUs, and PCRs with <500 reads were discarded for invertebrate OTUs. Finally, vertebrate sequences identified as livestock and domestic fowl (including pigs, cattle, sheep, goat, and chicken) were removed to retain only wild biota. We retained yak sequences since the wild yak occurs in the study region. A sediment sample in MCL (M8-S) was excluded after bioinformatic processing due to low read counts generated from this sample.

The qualitative data (incidence data) were in the form of a 0/1 matrix, with OTUs marked as 1 when they occurred at least once in three PCR replicates of a sample and marked as 0 when they occurred in none of the replicate PCRs. The quantitative data (abundance data) for each taxonomic group were obtained by first calculating the relative read abundance (RRA) of each OTU in a PCR result and then obtaining the average RRA across replicate PCRs for each sample. The RRA metric was calculated as the reads for each OTU divided by the total number of reads in that PCR.^106^

### Quantification and statistical analysis

#### α-diversity

Overlapping and unique OTU numbers for each taxonomic group were visualized using R package ggvenn.^107^ The taxa richness (Hill number of order *q* = 0), Shannon diversity index (Hill number of order *q* = 1), and Simpson diversity (Hill number of order *q* = 2) of each sample were calculated based on the RRA data using R package Hilldiv.^108^ The statistical significance for α- and β-diversity (see below) indexes between habitats was assessed using Wilcoxon rank-sum tests. To evaluate the effect of sampling effort on OTU detection, the iNEXT package^109^ was used to draw rarefaction and extrapolation curves of taxa richness, Shannon diversity, and Simpson diversity.

#### β-diversity

β-diversity indexes, including Jaccard (qualitative) dissimilarity and Bray-Curtis (quantitative) dissimilarity, were calculated to assess between-sample community compositional variation using the *beta.pair* and *beta.pair.abund* function in the R package betapart.^110^^,^^111^ We used principal coordinate analysis (PCoA) to visualize the dissimilarities among lakes and habitats (various water depths and sediment) using the *cmdscale* function in vegan. PCoA of cyanobacteria, diatoms, and invertebrates were based on Bray–Curtis dissimilarity matrixes, while PCoA of vertebrates was based on Jaccard dissimilarity matrixes, all of which were calculated using the *vegdist* function in the vegan package. Because the detection results revealed a wide range of aquatic, terrestrial, and aerial vertebrate species, and the pattern and amount of eDNA shedding could be highly variable among species, we determined that a qualitative measurement was more feasible than a quantitative one. The significance of community compositional differences between lakes, between habitats, and among water depths and horizontal locations (water columns) was further evaluated using the *adonis2* function in vegan and permutational multivariate analysis of variance (PERMANOVA) tests based on 999 permutations.

#### Characteristic taxa identification

To identify the taxa characteristic of different habitats, we conducted similarity percentage analysis (SIMPER) and Specificity-Occupancy analysis. SIMPER was performed based on RRA data using the *simper* function in vegan to calculate the contribution of individual OTUs to the overall dissimilarity between communities in different habitats. Specificity-Occupancy analysis was carried out for each OTU as described previously.^112^ Briefly, the specificity of OTU *S* in habitat *H* was defined as the ratio of the average abundance of *S* in all samples of *H* to the sum of the average abundance of *S* over all habitats. Occupancy was calculated as the number of samples in *H* where *S* occurred divided by the total number of samples in *H*. OTUs with ≥0.7 specificity and ≥0.7 occupancy were deemed as specialist taxa to a habitat type. The results of these analyses were compared to check their consistency.

#### Co-occurrence networks

Ecological networks consist of nodes (individual taxa) and links (correlations in occurrence/abundance) and can be used to infer ecological interactions.^59^^,^^113^^,^^114^ Co-occurrence networks of multitrophic communities in a habitat were inferred based on Spearman correlations (correlation coefficient |*r*| ≥0.75, adjusted *p* <0.05) between OTUs of the same and different taxonomic groups based on the RRA data,^23^ using the *corr.test* function in the R package psych.^115^ Cyanobacteria, diatoms, and invertebrates eDNA most likely reflect the occurrence of the corresponding taxa at the sampling sites, since living organisms themselves can be captured in the samples. In contrast, vertebrate eDNA is released from organisms and can disperse far from the site of origin. Additionally, the majority of our detected vertebrate species were terrestrial. Therefore, vertebrate OTUs were excluded from the co-occurrence network analysis. To increase the reliability of the inferred networks, only OTUs with an average RRA across all relevant samples ≥0.01% and detected in no less than 30% of the samples were incorporated.^24^ Network visualization was conducted in Gephi v.0.9.7,^93^ using the Fruchterman-Reingold placement algorithm. Network parameters describing node and topological properties, including connectedness (degree), weighted degree, complexity (linkage density), average path length, clustering coefficient, and modularity, were calculated in the R package igraph.^116^

Network cohesion is a metric that measures the degree of connectivity of microbial communities^26^ and has been shown to be a strong predictor of network stability.^67^ We calculated positive and negative cohesion values for each network based on correlation matrixes and the RRA data of the OTUs. The positive and negative connectedness values of each OTU were obtained by averaging the positive and negative correlations, respectively. The positive and negative cohesions of each sample were calculated by summing the positive and negative connectedness of each OTU multiplied by its relative abundance.^26^ The statistical significance of multiple-group comparisons was evaluated using Kruskal-Wallis tests, followed by pairwise comparisons using Wilcoxon rank-sum tests. *P*-values were Benjamini-Hochberg-corrected where applicable.^117^

To identify keystone nodes (i.e., OTUs) in the networks, two parameters were calculated: within-module connectivity (*Z*_*i*_) and among-module connectivity (*P*_*i*_). *Z*_*i*_ describes the extent to which node *i* is connected to other nodes in the same module, and *Pi* reflects the degree to which node *i* is connected to other modules.^118^ In this study, nodes were classified into four roles using commonly used parameter thresholds: network hubs (*Z*_*i*_ >2.5, *P*_*i*_ >0.62), module hubs (*Z*_*i*_ >2.5, *P*_*i*_ ≤0.62), connectors (*Z*_*i*_ ≤2.5, *P*_*i*_ >0.62), and peripherals (*Z*_*i*_ ≤2.5, *P*_*i*_ ≤0.62).^64^^,^^118^

All statistical analyses were performed in the R v.4.2.1 environment.^92^ The ggplot2 package was used for data visualization.^119^

## Supplemental information

Document S1. Figures S1–S4 and Tables S1–S8

Data S1. Sampling site information and water physiochemical parameters, related to the STAR Methods

Data S2. Summary of the detected taxonomic operational units (OTUs), related to Figure 1
