# Supplementary material for: Distinct multitrophic biodiversity composition and community organization in a freshwater lake and a hypersaline lake on the Tibetan Plateau
Source: iScience. 2024 May 27;27(6):110124. doi: 10.1016/j.isci.2024.110124 (PMC11217615; doi:10.1016/j.isci.2024.110124)
Supplement: Document S1. Figures S1–S4 and Tables S1–S8 [file mmc1.pdf]

iScience, Volume *27*

Supplemental information

Distinct multitrophic biodiversity composition and community organization in a freshwater lake and a hypersaline lake on the Tibetan Plateau

Si-Yu Zhang (张思煜), Qi Yan (闫琦), Jindong Zhao (赵进东), Yongqin Liu (刘勇勤), and Meng Yao (姚蒙)

Supplemental Tables

Table S1. Primer information and PCR programs, Related to STAR Methods.

Target group	Primer name	Gene region	Amplicon length (bp)	Primer sequence (5′−3′)	PCR program	Reference
**Cyanobacteria**	CYA359F<br>CYA784R	16S	386	GGGGAATYTTCCGCAATGGG<br>ACTACWGGGGTATCTAATCCC	95°C 10 min; 35 cycles of 95°C 15 s, 60°C 30 s, and 72°C 45 s; 72°C 5 min	Monchamp et al., 2018[1]
**Diatoms**	708F_2<br>R3_1	rbcL	263	AGGTGAAGTTAAAGGTTCATACTTDAA<br>CCTTCTAATTTACCAACAACTG	95°C 15 min; 35 cycles of 95°C 45 s, 55°C 45 s, and 72°C 45 s	Vasselon et al., 2017[2]
**Invertebrates**	BF1<br>BR2	COI	316	ACWGGWTGRACWGTNTAYCC<br>TCDGGRTGNCCRAARAAYCA	95°C 5 min; 40 cycles of 95°C 30 s, 50°C 45 s, and 68°C 45 s; 72°C 10 min	Elbrecht and Leese, 2017[3]
**Vertebrates**	Tele02-F<br>Tele02-R	12S	167	AAACTCGTGCCAGCCACC<br>GGGTATCTAATCCCAGTTTG	95°C 10 min; 45 cycles of 95°C 30 s, 63°C 30 s, and 72°C 30 s; 72°C 10 min	Taberlet et al., 2018[4]

Table S2. Bioinformatics processing of next-generation sequencing data, Related to STAR Methods. Numbers of sequences (reads and unique sequences) following corresponding procedures are shown.

**Procedure**	**Program**	**Parameter**	**Cyanobacteria**		**Diatoms**		**Invertebrates**		**Vertebrates**	
			**CRL**	**MCL**	**CRL**	**MCL**	**CRL**	**MCL**	**CRL**	**MCL**
Raw data (G)			12.8	10.0	11.1	11.7	17.7	15.1	9.6	10.2
Paired-end merging	obi alignpairedend	No. of reads	25,534,539	20,044,055	22,201,143	23,499,937	35,378,701	30,118,372	32,068,775	34,066,150
Alignment quality filtering	obi grep	Min. alignment score	0.7	0.7	0.75	0.75	0.7	0.7	0.8	0.8
		No. of reads	24,089,094	17,305,691	21,534,590	19,755,065	25,864,342	24,183,713	29,747,738	32,974,879
Sample assignment	obi ngsfilter	No. of reads	13,682,945	7,865,053	3,446,156	2,639,699	19,051,442	18,023,796	9,909,014	9,039,157
Dereplication	obi uniq	No. of unique seq.	4,772,664	4,391,207	450,938	630,355	8,424,835	6,920,821	694,169	544,318
Denoising	obi grep	Min. seq. length	250	250	200	200	250	250	100	100
		No. of reads	8,072,932	2,956,805	2,859,876	1,881,497	8,334,114	9,733,107	8,566,113	7,885,990
		No. of unique seq.	51,358	33,286	9,865	11,522	84,593	69,178	32,598	26,468
	obi clean	No. of reads	4,187,213	2,017,512	2,110,809	1,381,848	5,772,972	6,187,859	6,351,818	5,698,706
		Average reads/PCR	26,329	14,298	13,445	10,312	35,617	43,879	40,156	39,970
		No. of unique seq.	15,128	13,964	2,948	3,650	29,945	20,993	7,591	6,029
OTU clustering	Sumaclust	Similarity threshold	0.97		0.97		0.97		0.98	
		No. of preliminary OTUs	2,095		529		5,147		2,270	
Taxonomic assignment	Relevant databases	No. of OTUs	189		487		1,631		40	
Contamination removal	negative control counts subtraction	No. of OTUs	188		487		1,630		40	
	Tag- jump control	No. of OTUs	135		448		1,522		39	
PCR quality filtering		No. of OTUs	134		443		1,519		37	
Livestock/fowl removal		No. of final OTUs	134		443		1,519		28	

Table S3. Summary of the number of OTUs assigned at various taxonomic levels (at the finest level) within each group for Chang-re Lake (CRL), Mang co Lake (MCL), and all samples, Related to Figure 1.

Taxonomy	Cyanobacteria			Diatoms			Invertebrates			Vertebrates		
	CRL	MCL	All	CRL	MCL	All	CRL	MCL	All	CRL	MCL	All
Phylum	—	—	—	90	40	125	17	3	20	—	—	—
Class	22	6	26	36	65	100	1,048	212	1,239	—	—	—
Order	10	5	13	9	7	14	94	16	108	—	—	—
Family	28	9	35	21	16	34	12	8	19	—	—	—
Genus	39	31	58	113	50	153	28	94	119	9	9	11
Species	0	2	2	16	3	17	9	6	14	13	3	17
Total	99	53	134	285	181	443	1,208	339	1,519	22	12	28

Table S4. Results of PERMANOVA of OTU composition between Chang-re Lake (CRL) and Mang co Lake (MCL) and between samples within each lake, Related to Figure 4. Analysis was based on the quantitative Bray-Curtis index for cyanobacteria, diatoms, and invertebrates, and on the qualitative Jaccard index for vertebrates. Significant (<0.05) *p* values are shown in bold.

Group	Comparison			*F*	Df	R^2	*p*
Cyanobacteria (Bray-Curtis)	Among 10 habitats			8.392	9,79	0.489	**0.001**
	CRL vs MCL			8.922	1,87	0.093	**0.001**
	CRL	Water vs Sediment		84.266	1,51	0.623	**0.001**
		Water	Among depths	1.017	3,36	0.045	0.402
			Among columns	3.533	11,28	0.578	**0.011**
	MCL	Water vs Sediment		10.994	1,34	0.244	**0.002**
		Water	Among depths	1.603	3,25	0.143	0.190
			Among columns	1.352	10,18	0.402	0.188
Diatoms (Bray-Curtis)	Among 10 habitats			6.415	9,80	0.419	**0.001**
	CRL vs MCL			42.192	1,88	0.324	**0.001**
	CRL	Water vs Sediment		2.911	1,43	0.063	**0.007**
		Water	Among depths	0.954	3,28	0.064	0.517
			Among columns	2.139	11,20	0.524	**0.001**
	MCL	Water vs Sediment		3.542	1,43	0.076	**0.003**
		Water	Among depths	1.826	3,32	0.130	**0.028**
			Among columns	1.518	10,25	0.361	**0.025**
Invertebrates (Bray-Curtis)	Among 10 habitats			13.339	9,90	0.572	**0.001**
	CRL vs MCL			48.066	1,98	0.329	**0.001**
	CRL	Water vs Sediment		18.435	1,52	0.262	**0.001**
		Water	Among depths	1.454	3,36	0.069	0.072
			Among columns	3.022	11,28	0.526	**0.001**
	MCL	Water vs Sediment		36.660	1,44	0.455	**0.001**
		Water	Among depths	1.491	3,32	0.108	0.178
			Among columns	1.418	10,25	0.343	0.106
Vertebrates (Jaccard)	Among 10 habitats			2.269	9,55	0.271	**0.001**
	CRL vs MCL			13.048	1,63	0.172	**0.001**
	CRL	Water vs Sediment		1.171	1,45	0.025	0.315
		Water	Among depths	0.451	3,30	0.049	0.970
			Among columns	0.613	11,22	0.247	0.972
	MCL	Water vs Sediment		0.797	1,16	0.047	0.450
		Water	Among depths	0.883	3,13	0.165	0.562
			Among columns	1.030	9,7	0.577	0.481

Table S5. Contributions of various OTUs to community difference as indicated by SIMPER analysis, Related to Figure 4. Only OTUs with a contribution >5% are shown.

Group	Comparison	Habitat	OTUs	Contribution	SD
Cyanobacteria	CRL vs MCL	CRL	CYA_003 *Cyanobium*	16.79%	0.171
			CYA_006 *Cyanobium*	5.33%	0.112
		MCL	CYA_101 *Geitlerinema*	9.23%	0.164
	CRL	Water	CYA_003 *Cyanobium*	25.46%	0.151
	water vs sediment	Sediment	CYA_006 *Cyanobium*	20.40%	0.147
	MCL	Water	CYA_003 *Cyanobium*	29.74%	0.161
	water vs sediment	Sediment	CYA_101 *Geitlerinema*	24.42%	0.200
Diatoms	CRL vs MCL	CRL	DIA_081 *Gedaniella*	15.38%	0.176
			DIA_030 *Nitzschia*	8.38%	0.141
		MCL	DIA_300 *Nitzschia lembiformis*	8.76%	0.074
			DIA_275 *Pinnularia*	7.12%	0.092
			DIA_282 Bacillariophyta	6.58%	0.051
			DIA_297 Bacillariophyceae	5.88%	0.046
	CRL	Water	DIA_030 *Nitzschia*	10.94%	0.149
	water vs sediment		DIA_053 Bacillariophyta	6.02%	0.129
		Sediment	DIA_081 *Gedaniella*	21.96%	0.168
	MCL	Water	DIA_275 *Pinnularia*	7.67%	0.082
	water vs sediment		DIA_282 Bacillariophyta	5.67%	0.049
		Sediment	DIA_300 *Nitzschia lembiformis*	10.75%	0.104
			DIA_297 Bacillariophyceae	6.32%	0.049
			DIA_291 Bacillariophyta	5.56%	0.157
			DIA_279 Bacillariophyceae	5.14%	0.045
Invertebrates	CRL vs MCL	CRL	INV_0072 Insecta	13.74%	0.121
		MCL	INV_1289 Insecta	22.99%	0.140
			INV_1210 Insecta	6.39%	0.041
	CRL	Water	INV_0072 Insecta	18.47%	0.105
	water vs sediment		INV_0228 Copepoda	7.23%	0.095
	MCL	Water	INV_1289 Insecta	29.22%	0.082
	water vs sediment		INV_1210 Insecta	7.11%	0.034
		Sediment	INV_1248 *Artemia urmiana*	7.34%	0.092
			INV_1265 Flabellinia	7.29%	0.087
Vertebrates	CRL vs MCL	CRL	VER_16 *Bos grunniens*	21.92%	0.186
			VER_01 *Fulica atra*	5.39%	0.094
		MCL	VER_06 *Tadorna ferruginea*	12.18%	0.152
			VER_14 *Anas platyrhynchos*	9.57%	0.152
			VER_13 *Canis lupus*	8.56%	0.185
			VER_27 *Larus brunnicephalus*	7.34%	0.132
	CRL	Water	VER_16 *Bos grunniens*	23.19%	0.168
	water vs sediment		VER_23 *Anser*	8.01%	0.127
			VER_06 *Tadorna ferruginea*	8.00%	0.100
		Sediment	VER_05 *Mareca*	12.31%	0.160
			VER_01 *Fulica atra*	10.14%	0.136
	MCL	Water	VER_06 *Tadorna ferruginea*	10.74%	0.171
	water vs sediment		VER_14 *Anas platyrhynchos*	9.43%	0.165
			VER_13 *Canis lupus*	8.81%	0.196
			VER_27 *Larus brunnicephalus*	7.78%	0.139
			VER_16 *Bos grunniens*	7.15%	0.166
		Sediment	VER_04 *Ctenopharyngodon idella*	48.41%	0.001

Table S6. Characteristic OTUs of different habitats identified by Specificity-Occupancy analysis, Related to Figure 4.

Group	Comparison	Habitat	Characteristic OTUs	Specificity	Occupancy
Cyanobacteria	CRL vs MCL	CRL	CYA_004 Cyanobiaceae	0.973	0.830
			CYA_006 *Cyanobium*	0.922	1.000
			CYA_013 *Cyanobium*	0.946	0.774
		MCL	CYA_101 *Geitlerinema*	1.000	0.806
	CRL water vs sediment	Sediment	CYA_004 Cyanobiaceae	0.995	0.923
			CYA_006 *Cyanobium*	0.992	1.000
			CYA_013 *Cyanobium*	0.991	0.923
	MCL water vs sediment	Water	CYA_003 *Cyanobium*	0.742	1.000
			CYA_099 Cyanobacteriia	1.000	0.724
		Sediment	CYA_101 *Geitlerinema*	0.830	0.857
Diatoms	CRL vs MCL	CRL	DIA_004 *Nitzschia*	0.984	0.733
			DIA_017 Bacillariaceae	1.000	0.800
			DIA_030 *Nitzschia*	0.999	0.844
			DIA_081 *Gedaniella*	0.989	0.889
			DIA_101 *Staurosira*	1.000	0.733
		MCL	DIA_275 *Pinnularia*	0.989	0.956
			DIA_277 *Navicula*	0.962	0.978
			DIA_278 *Navicula*	0.989	0.978
			DIA_279 Bacillariophyceae	0.991	0.978
			DIA_281 *Nitzschia frustulum*	1.000	0.956
			DIA_282 Bacillariophyta	0.999	0.978
			DIA_288 Naviculaceae	0.914	0.978
			DIA_292 Naviculales	0.986	0.822
			DIA_294 Bacillariaceae	1.000	0.978
			DIA_297 Bacillariophyceae	1.000	0.978
			DIA_300 *Nitzschia lembiformis*	0.998	0.978
	CRL water vs sediment	Water	DIA_030 *Nitzschia*	0.804	0.813
		Sediment	DIA_017 Bacillariaceae	0.795	0.923
			DIA_032 *Navicula*	0.940	0.769
			DIA_050 *Anomoeoneis*	0.995	0.846
			DIA_103 *Halamphora*	0.987	1.000
			DIA_107 *Halamphora*	0.981	0.846
	MCL water vs sediment	Water	DIA_277 *Navicula*	0.919	1.000
			DIA_281 *Nitzschia frustulum*	0.875	0.972
			DIA_282 Bacillariophyta	0.747	1.000
			DIA_287 Catenulaceae	0.853	0.722
			DIA_292 Naviculales	0.929	0.833
		Sediment	DIA_279 Bacillariophyceae	0.736	0.889
Invertebrates	CRL vs MCL	CRL	INV_0019 Insecta	0.999	0.778
			INV_0023 Arachnida	1.000	0.870
			INV_0072 Insecta	1.000	0.889
			INV_0081 Arachnida	1.000	0.796
			INV_0117 Insecta	1.000	0.870
			INV_0130 Insecta	1.000	0.759
			INV_0163 Insecta	1.000	0.722
			INV_0177 Bivalvia	1.000	0.981
			INV_0228 Copepoda	1.000	0.759
			INV_0229 Arachnida	1.000	0.741
			INV_0236 Insecta	1.000	0.759
		MCL	INV_1202 Insecta	0.999	0.870
			INV_1208 Insecta	1.000	0.761
			INV_1210 Insecta	1.000	0.935
			INV_1248 *Artemia urmiana*	1.000	0.804
			INV_1285 Insecta	1.000	0.804
			INV_1289 Insecta	0.999	0.913
			INV_1298 Branchiopoda	1.000	0.761

			INV_1304 Hydrozoa	0.998	0.891
	CRL<br>water vs sediment	Water	INV_0019 Insecta	1.000	1.000
			INV_0047 Insecta	1.000	0.875
			INV_0052 Calcarea	1.000	0.825
			INV_0055 Scyphozoa	0.996	0.700
			INV_0072 Insecta	0.997	1.000
			INV_0077 Insecta	1.000	0.900
			INV_0081 Arachnida	0.852	0.900
			INV_0115 Anthozoa	1.000	0.925
			INV_0117 Insecta	0.965	0.975
			INV_0129 Gastropoda	1.000	0.925
			INV_0130 Insecta	0.996	1.000
			INV_0145 Insecta	1.000	0.875
			INV_0155 Haplosclerida	0.884	0.725
			INV_0163 Insecta	0.999	0.950
			INV_0190 Gastropoda	1.000	0.925
			INV_0229 Arachnida	0.997	0.975
			INV_0233 Insecta	1.000	0.700
			INV_0236 Insecta	0.994	0.975
			INV_0248 Insecta	1.000	0.750
			INV_0301 Insecta	1.000	0.750
			INV_0367 Arachnida	0.970	0.725
		Sediment	INV_0013 Hydrozoa	0.993	0.786
			INV_0039 Scyphozoa	0.801	0.714
			INV_0056 Asteroidea	1.000	0.786
			INV_0060 Arachnida	0.997	0.714
			INV_0098 Insecta	1.000	0.786
			INV_0109 Arachnida	0.988	0.714
			INV_0128 Arachnida	1.000	0.714
			INV_0133 Arachnida	1.000	0.857
			INV_0151 Anthozoa	0.997	0.857
			INV_0152 Hydrozoa	0.923	0.714
			INV_0177 Bivalvia	0.802	0.929
	MCL<br>water vs sediment	Water	INV_1202 Insecta	0.903	1.000
			INV_1208 Insecta	1.000	0.972
			INV_1210 Insecta	0.903	1.000
			INV_1285 Insecta	0.995	1.000
			INV_1289 Insecta	0.996	1.000
			INV_1297 Hydrozoa	1.000	0.778
			INV_1298 Branchiopoda	1.000	0.972
			INV_1304 Hydrozoa	0.991	0.972
		Sediment	INV_1204 Arachnida	0.886	0.800
			INV_1218 Hydrozoa	0.998	0.700
			INV_1235 Insecta	0.999	0.900
			INV_1248 *Artemia urmiana*	0.802	0.800
			INV_1265 Flabellinia	0.997	0.900
			INV_1266 Enoplea	1.000	0.700
Vertebrates	CRL vs MCL	CRL	VER_16 *Bos grunniens*	0.762	0.915

Table S7. Properties of multitrophic cooccurrence networks in different lakes and habitats, Related to Figures 5 and 6.

Network properties		CRL_water	CRL_sediment	MCL_water	MCL_sediment
Overall	Total nodes	12	83	35	95
	Total correlations	15	229	261	757
	Positive correlations	14	190	261	752
	Negative correlations	1	39	0	5
Node properties (Mean ± SE)	Connectedness (degree)	2.500 ± 0.417	5.518 ± 0.512	14.914 ± 1.516	15.937 ± 1.504
	Weighted degree	2.048 ± 0.337	4.553 ± 0.424	12.871 ± 1.341	14.115 ± 1.358
	Complexity (linkage density)	0.208 ± 0.035	0.066 ± 0.006	0.426 ± 0.043	0.168 ± 0.016
Topology	Average path length	1.789	2.982	1.129	2.121
	Clustering coefficient	0.529	0.511	0.867	0.778
	Number of modules	3	13	5	13
	Modularity	0.384	0.481	0.080	0.245
Cohesion (Mean ± SE)	Total cohesion	0.364 ± 0.014	0.466 ± 0.043	0.062 ± 0.007	0.585 ± 0.074
	Positive cohesion	0.069 ± 0.014	0.275 ± 0.031	0.062 ± 0.007	0.474 ± 0.076
	Negative cohesion	0.296 ± 0.000	0.191 ± 0.026	0 ± 0	0.112 ± 0.017
	Negative:Positive cohesion	30.930 ± 17.684	0.781 ± 0.125	0 ± 0	0.339 ± 0.097

Table S8. Keystone OTUs identified based on values of within-module connectivity (*Zi* >2.5) and among-module connectivity (*Pi* >0.62), Related to Figure 5.

Habitat	Network role	OTU	*Zi*	*Pi*
CRL sediment	Connector	DIA_067 Naviculaceae	0.283	0.648
		DIA_074 Naviculales	0.283	0.653
		DIA_099 *Fistulifera*	0.000	0.698
		DIA_120 Fragilariaceae	−2.077	0.625
		DIA_277 *Navicula*	0.755	0.622
		INV_0152 Hydrozoa	0.000	0.625
MCL sediment	Connector	DIA_281 *Nitzschia frustulum*	0.000	0.667

Supplemental Figures

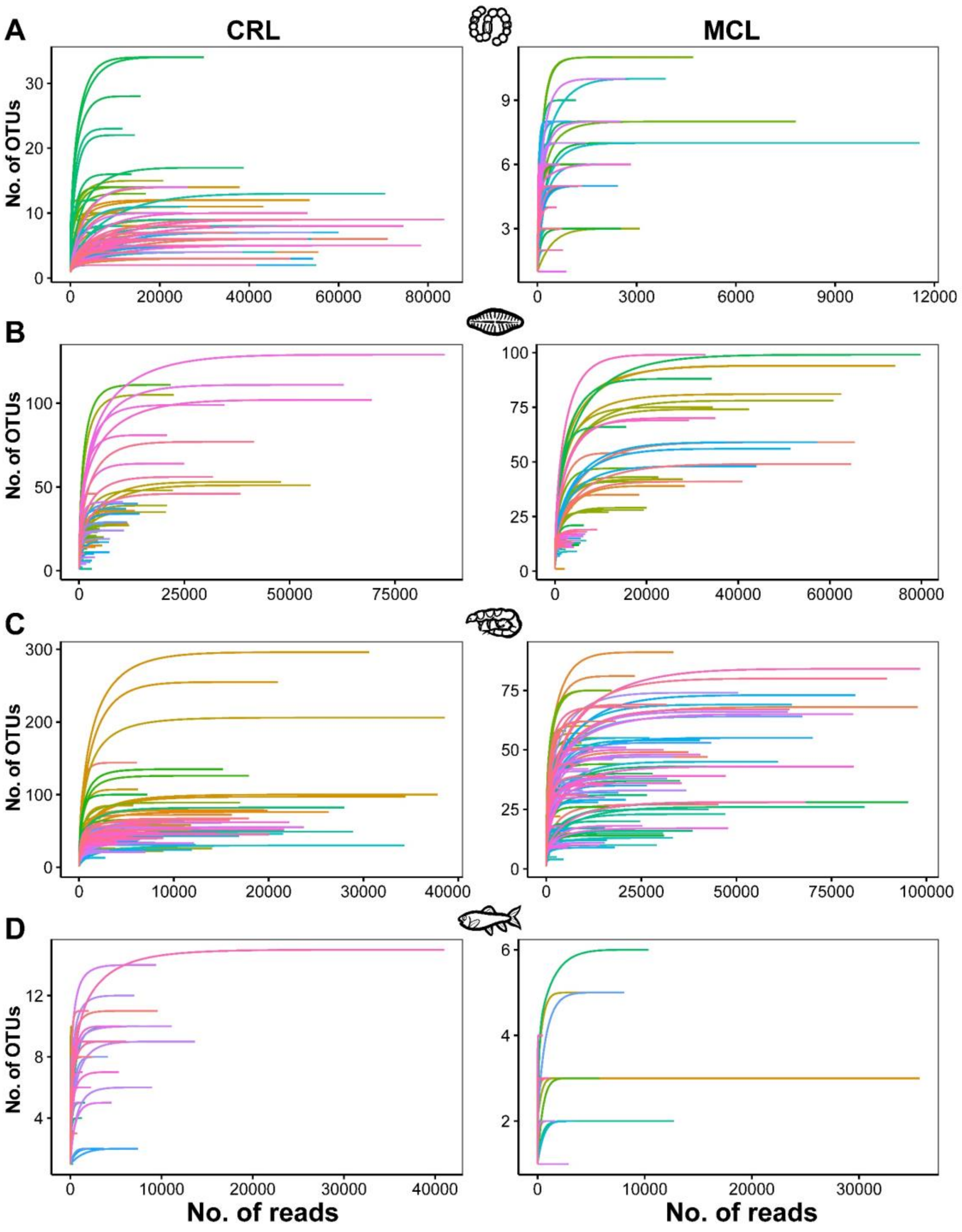

Figure S1. Rarefaction curves of OTUs detected with increasing sequence read counts, Related to STAR Methods. Results are shown for (A) cyanobacteria, (B) diatoms, (C) invertebrates, and (D) vertebrates in Chang-re Lake (CRL) and Mang co Lake (MCL). Each curve represents a PCR result.

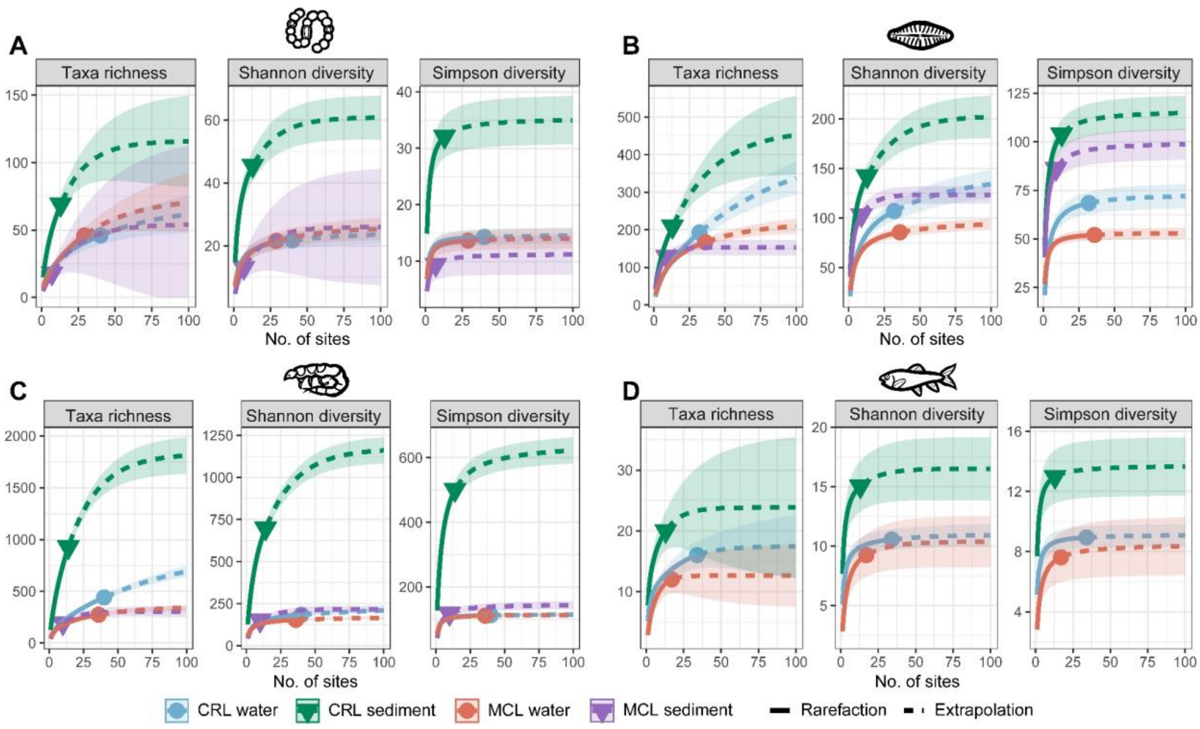

Figure S2. Rarefaction curves of OTU richness, Shannon diversity, and Simpson diversity of the four taxonomic groups detected with increasing number of sampling sites, Related to Figure 3. Results are shown for (A) cyanobacteria, (B) diatoms, (C) invertebrates, and (D) vertebrates in water and surface sediments from Chang-re Lake (CRL) and Mang co Lake (MCL).

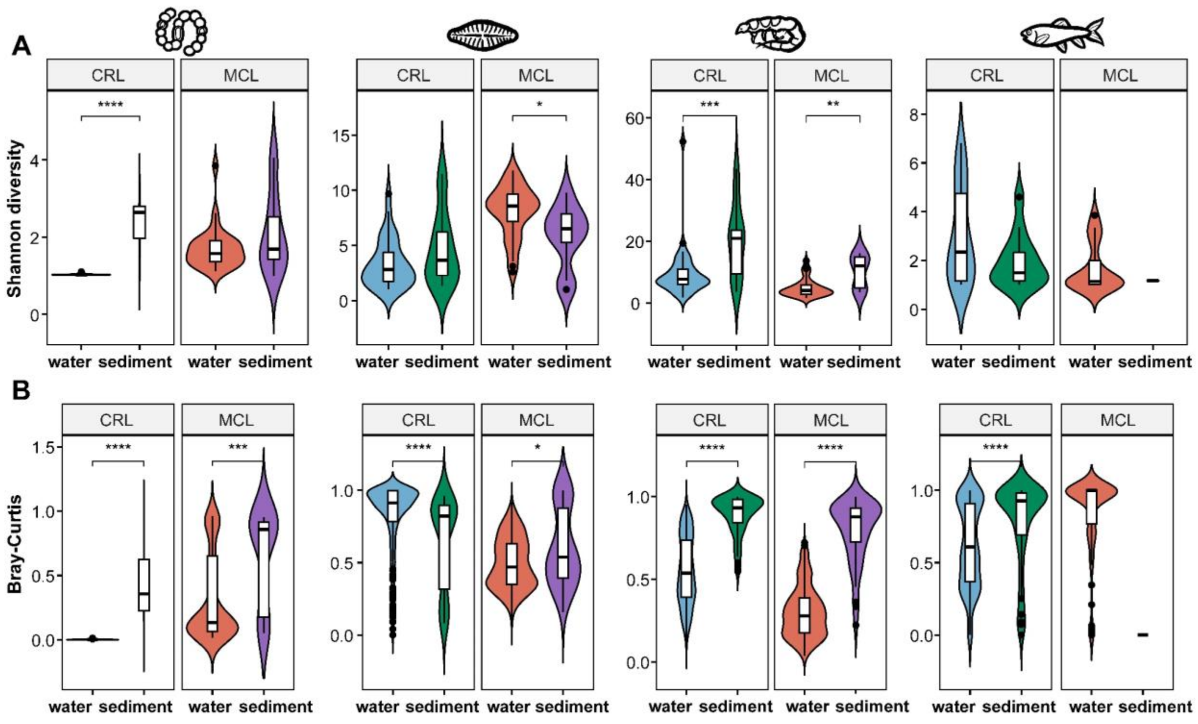

Figure S3. Comparisons of the α- and β-diversity of detected OTUs of the four taxonomic groups between water and sediment samples within Chang-re Lake (CRL) and Mang co Lake (MCL), Related to Figure 3. Results are shown for (A) Shannon diversity and (B) Bray-Curtis dissimilarity. Panels (left to right) show the results for cyanobacteria, diatoms, invertebrates, and vertebrates. Significant differences for pairwise comparisons determined by Wilcoxon rank-sum tests are shown: *, $p < 0.05$; **, $p < 0.01$; ***, $p < 0.001$; ****, $p < 0.0001$.

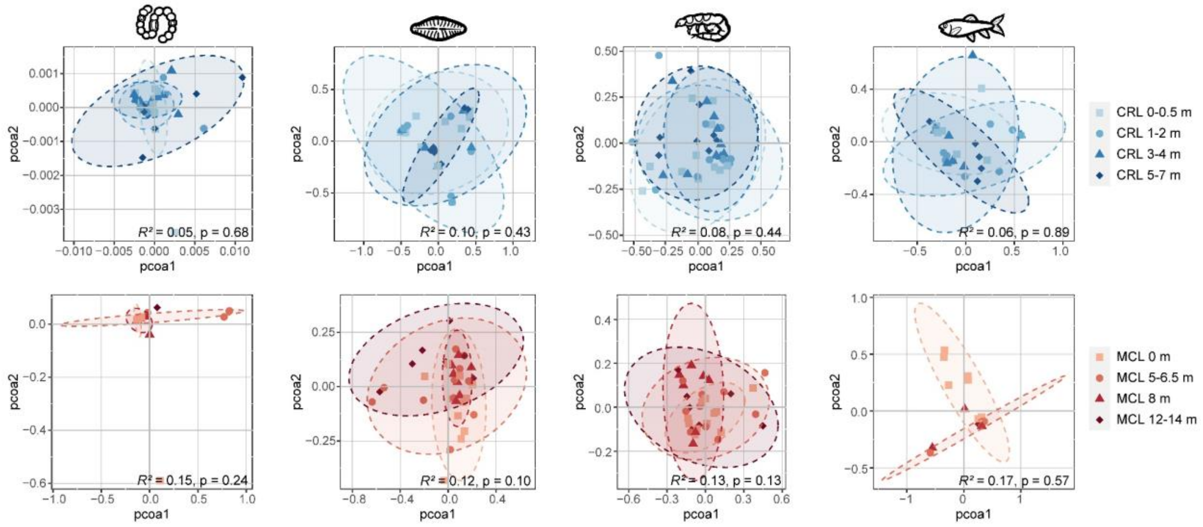

Figure S4. Principal coordinate analysis (PCoA) of the OTU compositions of the four taxonomic groups detected at various water depths from CRL (top panels) and MCL (bottom panels), Related to Figure 4. Panels (left to right) show the results for cyanobacteria, diatoms, invertebrates, and vertebrates. PCoA were based on the quantitative Bray-Curtis dissimilarity index for cyanobacteria, diatoms, and invertebrates, and on the qualitative Jaccard dissimilarity index for vertebrates. Results of PERMANOVA for the four depth groups are shown in each panel. See **Table S4** for more detailed results.

References

1. Monchamp, M.-E., Spaak, P., Domaizon, I., Dubois, N., Bouffard, D., and Pomati, F. (2018). Homogenization of lake cyanobacterial communities over a century of climate change and eutrophication. Nat. Ecol. Evol. *2*, 317-324. https://doi.org/10.1038/s41559-017-0407-0.

2. Vasselon, V., Rimet, F., Tapolczai, K., and Bouchez, A. (2017). Assessing ecological status with diatoms DNA metabarcoding: Scaling-up on a WFD monitoring network (Mayotte island, France). Ecol. Indic. *82*, 1-12. https://doi.org/10.1016/j.ecolind.2017.06.024.

3. Elbrecht, V., and Leese, F. (2017). Validation and development of COI metabarcoding primers for freshwater macroinvertebrate bioassessment. Front. Environ. Sci. *5*, 11. https://doi.org/10.3389/fenvs.2017.00011.

4. Taberlet, P., Bonin, A., Zinger, L., and Coissac, E. (2018). Environmental DNA: For Biodiversity Research and Monitoring (Oxford University Press).
